# Editorial: The Impact of Neurofilament Light Chain (NFL) Quantification in Serum and Cerebrospinal Fluid in Neurodegenerative Diseases

**DOI:** 10.3389/fnins.2022.915115

**Published:** 2022-05-05

**Authors:** Isabella Zanella, Hélène Blasco, Massimiliano Filosto, Giorgio Biasiotto

**Affiliations:** ^1^Department of Molecular and Translational Medicine, University of Brescia, Brescia, Italy; ^2^Clinical Chemistry Laboratory, Cytogenetics and Molecular Genetics Section, Diagnostic Department, ASST Spedali Civili di Brescia, Brescia, Italy; ^3^Laboratoire de Biochimie et Biologie Moléculaire, CHRU de Tours, Tours, France; ^4^Unité INSERM U1253, Université de Tours, Tours, France; ^5^Department of Clinical and Experimental Sciences, NeMO-Brescia Clinical Center for Neuromuscular Diseases, University of Brescia, Brescia, Italy

**Keywords:** neurofilaments, axonal injury, neurodegeneration, neurofilament light chains (NFL), biomarkers

Neurofilaments (NFs) constitute the main structural proteins of the cytoskeleton of neurons both in the central (CNS) and peripheral (PNS) nervous system and are abundantly assembled in large myelinated axons. NFs are composed of four subunits, the light (NFL), medium (NFM), and heavy (NFH) chains plus α-internexin in CNS or peripherin in PNS. Small amounts of NFs, particularly NFLs, may be released from axons into blood and cerebrospinal fluid (CSF) in healthy individuals and this release increases with age. More significant amounts are loosed upon traumatic brain injury, stroke and in several neuroinflammatory, and neurodegenerative conditions (Yuan et al., 2017). NFLs are a promising biomarker for neuronal degeneration and death, for monitoring disease progression and effectiveness of therapies and recent studies have demonstrated the potential in predicting outcome in presymptomatic subjects at risk for neurological diseases, although the major issue is that NFLs seem not specific of a particular neuropathology (Gaetani et al., 2019; Thebault et al., 2020).

The aim of this Research Topic was to provide an updated overview on the potential of serum/CSF NFL quantification in diagnosing and monitoring neurodegenerative disorders. Several researchers contributed interesting point of views, focusing on distinct diseases and covering several important technical and clinical aspects.

Yuan and Nixon summarized the neuropathological basis of NFs as biomarkers of neurological injury or neurodegeneration. The authors focused their attention on mechanisms of NF release, their trafficking between brain and blood and major determinants of NF levels in CSF and blood. They reviewed their importance as biomarkers both in human neurological diseases and injuries and in animal models of these conditions, paying attention on crucial issues: the identity and forms of NF proteins detected by commonly used technologies, the most recent technological advances in their reliable detection and the urgent need of measurement standardization.

While CSF and blood NFLs are mostly used to monitor the severity degree of neurological diseases and the efficacy of therapies, recent evidence has highlighted the importance of their measurement in the identification of neurodegenerative diseases in their presymptomatic phases. Gaetani et al. summarized evidence in multiple sclerosis (MS), Alzheimer's disease (AD), frontotemporal dementia (FTD), and amyotrophic lateral sclerosis (ALS). This is a very important topic, considering that disease-modifying drugs are available for the treatment of diseases like MS and their efficacy often relies on early diagnosis, also bearing in mind that the study of the presymptomatic phases of neurological diseases is fundamental in understanding their early pathological mechanisms.

NFL release increases with age as well as in some pathologies, characteristic of old age, but little is known about how other comorbidities or physiological factors may affect this release. In their original article, Polymeris et al. investigated the association of estimated glomerular filtration rate (eGFR) and body mass index (BMI) with serum NFL in a large cohort of elderly patients with atrial fibrillation. In a multivariable model adjusted for all clinical variables, eGFR and BMI showed strong inverse association with NFL levels and in these associations both eGFR and BMI interacted with age. Age, eGFR and, to a lesser extent, BMI alone or all combined explained a significant proportion of NFL level variance. This study, providing crucial information on NFL homeostasis, places emphasis on how renal function and BMI contribute to NFL levels in the elderly, suggesting considering these and further physiological factors in NFL measurement interpretation.

Four manuscripts focused on NFL measurement in MS, perhaps the most popular application of this biomarker. Serum NFL levels correlate with disease activity and treatment efficacy, are predictive of poorer clinical outcomes, are elevated years before the clinical onset and in clinically isolated syndromes are predictive of conversion to clinically definite MS. In their review, Thebault et al. summarized important issues relative to analytical and clinical validity of serum NFL measurement, fundamental issues for their routine clinical use together with the currently applied tools to diagnose and monitor MS. The authors focused on preanalytical and assay standardization and data analysis methodologies and highlighted the main physiological determinants of serum NFL levels, possible confounding effects of comorbidities, the presence of anti-NFL antibodies, the lesion location, and physiologic kinetics of NFL distribution and clearance.

Ferreira-Atuesta et al. further emphasized the correlation between axonal damage and loss with progression and disability in MS, highlighting the pros and cons of NFLs as biomarker in MS. They thoroughly illustrated the correlation between NFL levels and clinical and radiological findings in MS, also considering the potential use of the biomarker in MS mimics. The authors also extensively discussed the currently used technologies, the correlation between NFL measurements in CSF and blood and the need of optimal and sensitive cut-off values. Finally, they interestingly focused on the influence of coexisting peripheral nerve and CNS diseases and antibodies raised against NFL on reliable measurements and clinical interpretation of NFL levels, also looking at other biomarkers to be examined independently or in relation to NFLs.

Pukoli et al. reported on the relationship between neuroactive metabolites, produced in the kynurenine pathway (KP) and activated by several pro-inflammatory cytokines, and the pathomechanisms of MS. The authors described how some neuroactive KP metabolites, produced by microglia and macrophages, may have a role in MS development, producing free radicals in the presence of redox active metals like Fe^2+^; activating N-methyl-D-aspartate receptors, resulting in excitotoxicity; inhibiting the re-uptake of glutamate by astrocytes resulting in neurotoxicity; and decreasing glutamine synthetase activity so limiting the recycling of glutamate to glutamine in astrocytes. Several studies confirmed the activation of KP in MS: during the early phase of MS the production of neuroprotective kynurenine metabolites counteracts the effects of neurotoxic metabolites, while during disease progression the excess of neurotoxic metabolites contributes to the progression of MS. Interestingly, kynurenine neurotoxic metabolites play a central role in axonal damage also through the destabilization of cytoskeleton by causing hyperphosphorylation of proteins like NFL and a positive association between KP metabolites and plasma NFL levels has been demonstrated.

In their original article, Masvekar et al. tried to develop and validate a CSF-biomarker-based molecular surrogate representing MS lesional activity, mediated by immune cells migrating from the periphery to the CNS and commonly reflected by contrast-enhancing lesions (CELs) on magnetic resonance imaging (MRI). They analyzed CSF and serum samples for 20 inflammatory and axonal damage biomarkers. The authors interestingly found a significant association of some biomarkers and NFL levels in CSF with lesional activity. IL12p40 and CHI3L1 seemed reproducibly the best CSF biomarkers of MS lesional activity. Though serum NFL levels were correlated with CSF NFLs, serum NFL measurement did not differentiate between non-active and active MS lesional inflammatory activity subgroups and were weakly correlated with number of CELs and pro-inflammatory biomarkers associated with lesional inflammatory activity.

Saak et al. explored the hypothesis that elevated serum NFL may suggest nervous system involvement in patients with primary myopathies. They determined serum NFL levels in patients with myotonic dystrophy type I (DMI) and II (DMII), mitochondrial diseases or facioscapulohumeral muscular dystrophy (FSHD), also including a control group of patients with genetic defects exclusively expressed in muscle. Finding significatively elevated levels of serum NFL in DMI, DMII and mitochondrial disease patients, the authors demonstrated that serum NFL levels may be used as biomarker of neuronal damage in muscle diseases with established nervous system involvement. They interestingly further showed that serum NFL were also raised in FSHD patients, for whom the involvement of the nervous system is not usually clinically apparent, suggesting serum NFL as a biomarker for neuronal damage in primary neuropathies.

A cohort of patients affected by movement disorders with nigrostriatal neurodegeneration were studied by Diekämper et al. using DaTscan SPECT. In these patients there was a strong correlation between NFL and plasma NFH levels and the changes of presynaptic dopamine transporter density in the pathological conditions involving putamen concomitant to nigrostriatal degeneration. Therefore, NFL concentration could help to understand the degree of impairment of motor functions also in Parkinson's disease.

The review of Zanardini et al. explored the possibility that exosomes could be correlated to NFL and the main proteins involved in FTD. NFL and exosome dosages could be important in genetic FTD and might be useful mainly before the clinical onset allowing to anticipate the therapeutic treatments.

The importance of NFL in ALS and FTD was reviewed by Verde et al. CSF NFL levels correlated positively with disease progression and negatively with survival in ALS. In FTD, NFL were more elevated than in healthy people and slightly more elevated than in other dementias. The NFL level in CSF correlates with the disease progression, but the differences seem not so important to justify a diagnostic utility. The authors underlined the importance to dose NFL in blood and described the recent advances which showed longitudinal kinetics in presymptomatic patients carrying causative mutations for ALS and FTD. Moreover, NFL could be important in ALS patients as pharmacodynamic biomarker in therapeutic clinical trials.

An interesting research was proposed by Dreger et al. to better define the NFL potential as biomarker in ALS. The authors applied the D50 progression model to overcome the heterogeneity of clinical presentation in patients. Enrolled patients were divided in three groups characterized by high, intermediate and low disease progression on the basis of D50 values. A significant difference and positive correlation between CSF NFL levels and disease activity were found comparing the groups. This model can therefore be recommended in future studies as a useful tool.

The clinical and biological implications of NFL dosage in rapidly progressive dementias were reviewed by Abu-Rumeileh and Parchi, focusing on prion and Creutzfeldt–Jakob diseases. NFL levels showed interesting prognostic and diagnostic performances and may be used as biomarker to predict clinical onset in PRNP (prion protein) mutation carriers. In addition, authors gained attention on the follow-up of cerebrovascular diseases. Then, the quantification of NFL in CSF and in blood could be a very useful tool in precision medicine also applied to rapidly progressive dementias.

The use of NFL measurement in Friedreich ataxia (FRDA) was deepened in the review of Frempong et al. Although serum NFL result higher in FRDA patients than in controls, they do not correlate with genetic and clinical severity, like GAA repeat length or disease progression and were paradoxically higher in young patients, decreasing with age as the pathology progresses. These evidences make difficult the use of NFL as a biomarker in FRDA. The authors proposed some hypotheses to explain the anomalous NFL kinetics in FRDA patients, like a relatively large early loss of peripheral axons not contributing to clinical progression or the reflection of other components of the pathophysiology of FRDA like abnormal lipid metabolism and lipid peroxidation.

The possible role of serum NFL levels in children with epileptic or febrile seizures was explored in the research article of Evers et al. NFL levels were studied comparing the results of both cohorts with those obtained in a further control cohort of children with febrile infections without convulsion. The results of the study evidenced that NFL levels did not reveal significant differences among the three cohorts of analyzed patients. Applying multivariate analysis, age was the best predictor of NFL levels followed by sex and C reactive protein. The study demonstrated an age-dependent decrease of NFL levels from early childhood until school age.

The extent of neurodegeneration is monitored by challenging clinical measures and MRI in Wolfram syndrome. The Research Topic of Eisenstein et al. tested the possibility to use NFL as biomarker especially in patients with MRI contraindications. The NFL levels were compared in children, young patients and controls in relation to the clinical severity and chosen brain region volumes. Increased NFL levels were related to worse smell identification, color vision and visual acuity. Higher NFL values were also correlated to smaller thalamic and brainstem volume and faster annual rate of decrease in thalamic volume during time. Therefore, NFL dosage could be a useful biomarker to follow the neurodegenerative process in this type of patients.

In conclusion, many questions are still open to better understanding the role of NFL levels in traumatic brain injury, stroke, neuroinflammatory and neurodegenerative conditions. This Research Topic of articles raises the most important issues to be considered in translating research findings in clinical practice.

## Author Contributions

All authors listed have made a substantial, direct, and intellectual contribution to the work and approved it for publication.

## Conflict of Interest

The authors declare that the research was conducted in the absence of any commercial or financial relationships that could be construed as a potential conflict of interest.

## Publisher's Note

All claims expressed in this article are solely those of the authors and do not necessarily represent those of their affiliated organizations, or those of the publisher, the editors and the reviewers. Any product that may be evaluated in this article, or claim that may be made by its manufacturer, is not guaranteed or endorsed by the publisher.
